# Cytotoxic Effects of Different Extracts and Latex of *Ficus carica* L. on HeLa cell Line

**Published:** 2011

**Authors:** Ghadam Ali Khodarahmi, Nasrollah Ghasemi, Farshid Hassanzadeh, Marzieh Safaie

**Affiliations:** a *Department of Medicinal Chemistry, Faculty of Pharmacy, Isfahan University of Medical Sciences, Isfahan, Iran. *; b *Department of Pharmacognosy, Faculty of Pharmacy, Isfahan University of Medical Sciences, Isfahan, Iran.*; c *Isfahan Pharmaceutical Sciences Research Center, Faculty of Pharmacy, Isfahan University of Medical Sciences, Isfahan, Iran.*

**Keywords:** *Ficuscarica*, HeLa, Cytotoxicity, Extraction

## Abstract

It has been reported that latex and extracts of different species of *Ficus* are cytotoxic to some human cancerous cell lines. In this study, cytotoxicity of fruit and leaf extracts as well as the latex of *Ficuscarica* L. on HeLa cell line were evaluated. ethanolic extracts of leaves and fruits were prepared through percolation and ethyl acetate and dichloromethane extracts were prepared by reflux method. Cytotoxic effects of these extracts and latex against HeLa cell line were then examined. Briefly, He Lacells were seeded at 2 × 10^4^ cells/mL in 96-well plates. After 24 h incubation at 37^°^C, the cells were treated with different concentrations of the extracts or latex. The viability of the cells was determined by the reduction of 3-(4, 5-dimethylthiazol-2-yl)-2, 5-diphenyl tetrazolium bromide (MTT) from formazan following 48 h incubation and the absorbance was measured at 540 nm using an ELISA plate reader. The results indicated that the latex and different extracts of *Ficus carica* could reduce the viability of the He Lacells at concentrations as low as 2 µg/mL in a dose dependent manner. The approximate IC_50_ values of the ethanolic, ethyl acetate and dichloromethane extracts of the leaves and fruits were 10, 19, 12 µg/mL and 12, 12, 11.5 µg/mL, respectively. The IC_50 _for the latex was about 17 µg/mL.

## Introduction

In recent years, there has been a global trend towards the use of natural phytochemicals present in natural products such as fruits, vegetables, and their extracts. *Ficus spp* (Fig) products are among the most important examples of natural products which have been widely used both as afood and as a medicine. 

In traditional medicine, the Fig roots are used in the treatment of leucoderma and ringworms and its fruits have antipyretic, purgative and aphrodisiac properties and have shown to be useful in inflammations and paralysis (1, 2).

The leaves are being used traditionally in the treatment of jaundice in some parts of India (3). 

The leaf extracts of *Ficus racemosa *(4) and *Ficus hispida *(5) possess significant hepatoprotective activity against carbon tetrachloride and paracetamol induced hepatotoxicity in rats. The methanol extract of the leaves of *Ficus carica* was recently shown to possess hepatoprotective activity in rats with liver damage induced by carbon tetrachloride at an oral dose of 500 mg/Kg by lowering the serum levels of aspartate aminotransferase, alanine aminotransferase, total serum bilirubin, and malondialdehyde equivalent, an index of lipid peroxidation of the liver (3). 

Fig tree latex was used traditionally to treat wart (6). The Fig tree latex therapy of warts was recently compared with cryotherapy which offers several beneficial effects including short duration therapy, no reports of any side-effects, ease-of-use, patient compliance, and a low recurrence rate (7) .The latex offers some other therapeutic effects such as anti-Herpes Simplex Virus (HSV)-1 (8), anthelmintic (9), anti mutagenic (10), antioxidative (11) and cytotoxic (12) activities. 

Chemical examination of *Ficus spp.* have shown the presence of psoralen, bergapten, umbelliferone (13,14), *β*-sitosterol, campesterol, stigmasterol, fucosterol, fatty acids (15), 6-(2-methoxy-Z-vinyl)-7-methyl-pyranocoumarin and 9,19-cycloarlane triterpenoid (16,17), 6-O-acyl-*â*-D-glucosyl- and 6-O-acyl-*β*-D-glucosyl- *β*-sitosterol (12), and lupeol acetate (18).

More recent investigations indicated that different parts of *Ficu*s species like fruit, stem, leaf and latex contain active ingredients like triterpenoides which have antioxidant and cytotoxic activities (19, 20, 21). For instance, triterpenes which were isolated from the aerial roots of *Ficusmicrocarpa* were cytotoxic to HONE-1, KB, and HT29 cell lines with IC_50_ values about 4.0-9.4 µM (22). In this study, cytotoxic activity of fruit and leaf extracts and the latex of *Ficus carica* L. on HeLa cell line were evaluated.

## Experimental

*Materials*

The plants were collected from the *Ficus*Garden of Isfahan University of Medical Sciences and identified as *Ficus carica. *A sample (herb. no 1778) was kept in the herbarium of the department of pharmacognosy. The fruits and leaves were air dried and the latex was kept at 4°C, before use. 

Tripan blue, 3-(4, 5-dimethylthiazol-2-yl)-2, 5-diphenyl tetrazolium bromide (MTT) and dimethyl sulfoxide (DMSO) were purchased from Merck (Merck, Germany). HeLa (human black cervix carcinoma, epithelioid) cells were purchased from Pasteur Institute of Iran (in Tehran). Roswell Park Memorial Institute (RPMI)-1640 culture medium, Fetal Bovine Serum (FBS), or Fetal Calf Serum (FCS), sodium pyruvate, penicillin/ streptomycin and trypsin-EDTA were purchased from Gibco (Gibco, Scotland). The absorbance was measured at 540 nm with an ELISA plate reader (Starfinn 2100, USA). 

*Extraction methods*

Ethanolic extracts of the fruits and the leaves were prepared by percolation with 70^°^ aqueous ethanol while ethyl acetate and dichloromethane extractions were conducted by reflux method. The solvents evaporated using a rotary evaporator. The dry extracts were then used for cytotoxic assays. The latex was used directly without any extraction or further purifications. 

*Sample and culture medium preparations*

HeLa cells were grown in RPMI1640 medium. Each 500 mL of the medium consisted of 5.2 g RPMI powder, 1 g of sodium bicarbonate, 5 mL of sodium pyruvate (1 mM), 5 mL of L-glutamine (2 mM), 2.5 mL of penicillin (10000 IU/mL) and 2.5 mL of streptomycin (10 mg/mL) supplemented with 50 mL heat-inactivated fetal calf serum (FCS) in deionized water (24). The completed media was sterilized by filtering through 0.22 µ microbiological filters. The pH of the medium was then adjusted to 7.4 using concentrated HCl or NaOH and kept at 4°C before use. Cell lines were maintained in a humidified atmosphere of 5% CO_2_ - 95% air at 37°C. 

The stock solutions (2000 µg/mL) were prepared by dissolving 20 mg of the dry extracts and the latex in 500 µL of DMSO followed by the addition of RPMI medium to 10 mL total volume. The stock solutions were then appropriately diluted with the medium and finally 20 µL of each dilution was added to the 96-well microplate containing 180 µL of the cell suspensions in order to reach 2, 5, 10, 20, 50, and 100 µg/mL final concentrations. Taxol was used as a positive control at 21.5 µg/mL (0.25 µM) final concentration in the wells.

*In-vitro cytotoxicity assay*

The cytotoxic effects of different extracts and the latex of *Ficus carica* L. against HeLa cells were determined by a rapid colorimetric assay using MTT. The results were compared with untreated control (25). In this assay, mitochondrial succinic dehydrogenase enzyme of viable cells would metabolically alter the yellow soluble MTT salt into a blue insoluble formazan product. The blue solid could be dissolved in DMSO and measured spectrophotometrically (26). 

Briefly, after 2-3 subcultures, 180 µL of the cells (5 x 10^4^ cells per mL of media) were seeded in 96-well microplates and incubated for 24 h (37°C, air-humidified 5% CO_2_). Then, 20 µL of different concentrations of the samples were added and the microplates were further incubated for 48 h at the same condition. Taxol was used as a positive control. The first column of the microplate containing 180 µL of the cell suspension and 20 µL RPMI was regarded as negative control while the blank wells consisted of only 200 µL the RPMI medium. To evaluate the cell survival, each well was then incubated with 20 µL of MTT solution (5 mg/mL in phosphate buffer solution) for 3 h. Afterwards, the media in each well was gently replaced with 200 µL DMSO and pipetted up and down to dissolve the formazan crystals. The absorbance of each well was measured at 540 nm using an ELISA plate reader. Standard curves (absorbance against the number of cells) for HeLa cell line was constructed and used for the calculation of percent cell survival. The percent cell survival was taken as 100% for the negative control. The percentage of cell viability was calculated using the following formula:

$\mathrm{\%Survival}=\frac{\left(\mathrm{Mean Abs.of the samples}-\mathrm{Mean Abs.of the blank}\right)}{\left(\mathrm{Mean Abs.of the negative control}-\mathrm{Mean Abs.of the blank}\right)}\times 100$

*Statistical analysis*

The results are the mean of three triplicate experiments. SIGMASTAT (Jandel Software, San Raphael, CA) was used for statistical analysis. Analysis of variance (ANOVA) was used to see the differences amongst various groups. Significance was assumed at p < 0.05.

## Results and Discussion

**Figure 1:**
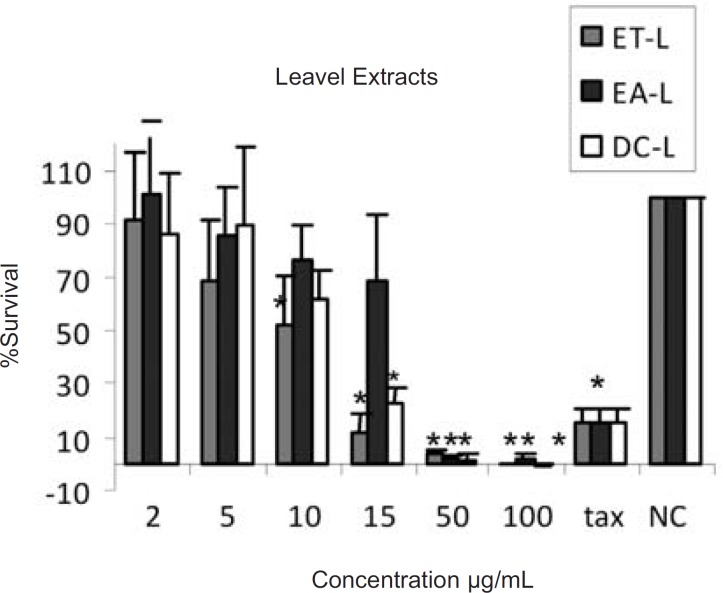
Cytotoxic effects of ethanol (ET-L) , ethyl acetate (EA-L) and dichloromethane (DC-L) extracts of the leaves of Ficus carica on HeLa cells, following exposure to different concentrations of the extracts, cell viability was assessed using the MTT method. Data are presented as mean±SD, *=p<0.05, n=9,tax=Taxol 21.5 µg/ml , NC = Negative control

**Figure 2:**
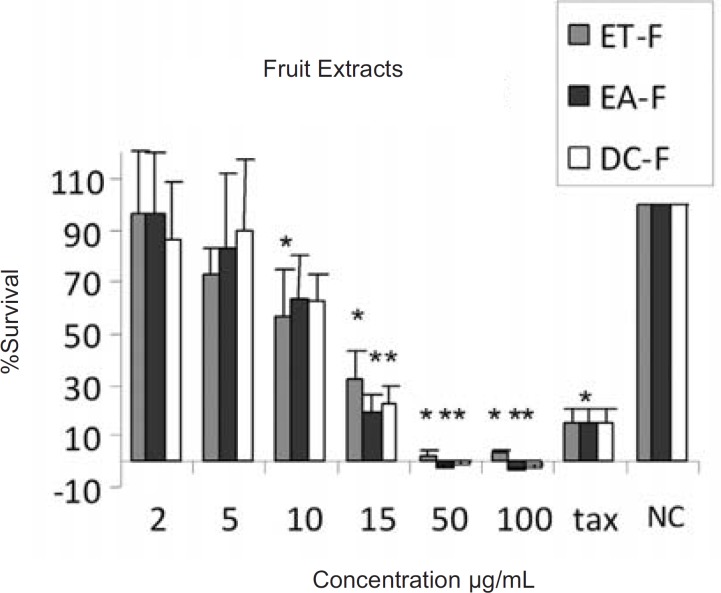
Cytotoxic effects of ethanol (ET-F) , ethyl acetate (EA-F) and dichloromethane (DC-F) extracts of the fruits of Ficus carica on HeLa cells, following exposure to different concentrations of the extracts, cell viability was assessed using the MTT method. Data are presented as mean±SD, *=p<0.05, n=9,tax=Taxol 21.5 µg/ml , NC = Negative control

**Figure 3:**
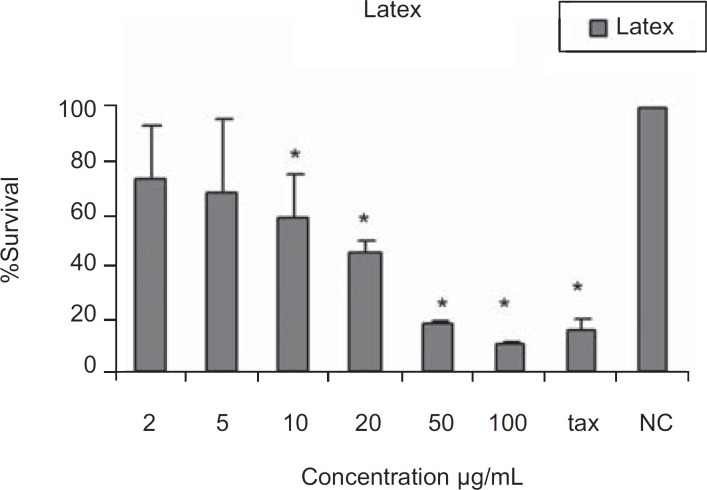
Cytotoxic effects latex of *Ficus carica *on HeLa cells Following exposure to different concentrations of the latex, cell viability was assessed using the MTT method. Data are presented as mean ± SD; * = p < 0.05; n = 9; tax = Taxol 21.5µg/ml , NC = Negative control

The results of cytotoxic assays of different extracts of the leaves, fruits and the latex of *Ficus carica *are shown in Figures 1, 2 and 3, respectively. The approximate concentrations of the extracts to reduce viability of the cells to about 50% ( IC_50_ ) is shown in Table 1.

**Table 1:** Approximate IC5 values of the extracts and latex on HeLa cellline

**Plant part**	**Extraction solvent**	**IC**_50_ **(µg/mL)**
Leaves	Ethanol	10
	Ethyl acetate	19
	Dichloromethane	12
Fruits	Ethanol	12
	Ethyl acetate	12
	Dichloromethane	11
Latex	Crude latex	17

The first scientific research on the cytotoxic activities of *Ficus* latex was performed by Ulman *et al.* (27). The cytotoxic effects may reside mostly in the resin of the latex of *Ficus* species, while the other ingredients of different parts of the plant may also contribute to these activities.

In the present work, cytotoxic effects of ethanol, ethyl acetate and dichloromethane extracts of *Ficus carica* and the crude latex were evaluated. According to the presence of different active ingredients in *Ficu*s spp. with diverse solubilities, it seems that the ethanolic extracts may contain more polar ingredients like carbohydrates, proteins, resins and glucosyl-sitosterols (21, 22, 23) while the less polar solvents, ethyl acetate and dichloromethane, could extract less polar materials like triterpenoids, present in the leaves and fruits, some of which have cytotoxic potentials (12). The results indicated that all extracts from the fruits, leaves and the latex have moderate cytotoxicity against HeLa cell line, without considerable difference, with approximate IC_50_ values of 10-20 µg/mL as shown in Table 1. This could be due to the partial solubility of different active ingredients in the employed solvents. 

These results are almost in accordance with the results obtained by Chiang *et al.* who separated some cytotoxic triterpenes from the aerial roots of *Ficus microcarpa *using moderate polarity solvent systems. The separated triterpenes werecytotoxic to HONE-1, KB and HT29 having IC_50 _values in the range of 4-10 µM (about 2-5 µg/mL) (22). On the other hand, 6-O-acyl-*β*-D-glucosyl- *β*-sitosterols which were provided by successive extractions with different polar solvents were also cytotoxic to a panel of cell lines including MCF-7 and Raji cells with approximately 25 µg/mL IC_50_ values (12).

The similar cytotoxic effects of the extracts and the latex could be due to the use of crude latex in our studies.
